# Influence of surface preparation on fracture load of resin composite-based repairs

**DOI:** 10.4317/jced.52084

**Published:** 2015-02-01

**Authors:** Rubén Agustín-Panadero, Rocío Mateos-Palacios, Juan-Luis Román-Rodríguez, María-Fernanda Solá-Ruíz, Antonio Fons-Font

**Affiliations:** 1 Associate Lecturer, Department of Dental Medicine, Faculty of Medicine and Dentistry, University of Valencia, Spain; 2 Master of Buccofacial Prosthetics, Department of Dental Medicine, Faculty of Medicine and Dentistry, University of Valencia, Spain; 3 Assistant Lecturer, Department of Dental Medicine, Faculty of Medicine and Dentistry, University of Valencia, Spain; 4 Senior Lecturer, Department of Dental Medicine, Faculty of Medicine and Dentistry, University of Valencia, Spain

## Abstract

The purpose of the present study is to evaluate the fracture load of composite-based repairs to fractured zirconium oxide (Z) crowns and to ceramic-fused-to-metal (CM) crowns, comparing different mechanical surface preparation methods. A total of 75 crowns were repaired; samples then underwent dynamic loading and thermocycling. Final fracture load values for failure of the repaired crowns were measured and the type of fracture registered. Group I: CM: Surface preparation with a diamond bur + 9.5% Hydrofluoric Acid (HF) etching; Group II): CM: air-particle (Al2O3) + 9.5% HF; Group III: CM: Silica coating (SiO2); Group IV): Z: air-particle (Al2O3) + HF 9.5%; Group V) Z: Silica coating (SiO2). Of the three CM groups, Group I (CM-diamond bur) showed the highest mean failure value, with significant difference in comparison with Group III (CM-silica coating). For the zirconia groups, the highest value was obtained by Group V (silica coating).

** Key words:**Crown, ceramic-fused-to-metal, zirconia, resin-composite, ceramic covering.

## Introduction

Nowadays, dental restorations with crowns represent a significant proportion of dental treatments. In spite of the high predictability and high success rates of ceramic-fused-to-metal crowns, approximately 95.9% over ten years (1) and an 88.8% success rate over five years for all-ceramic crowns (2), it is inevitable that these restorations occasionally suffer problems that require their repair or replacement. The use of composite resin-based repair systems has been proposed as an alternative therapy whenever fractures of the covering ceramic occur. This repair option offers various advantages, mainly of speed, low cost, and ease-of-use (3,4).

-Purpose: To measure the final fracture values of repairs carried out with resin composite to adhesive fractures of the covering ceramic, exposing the cores of zirconium oxide (Z) crowns and ceramic-fused-to-metal (C) crowns, comparing different surface preparation methods.

## Material and Methods

Seventy-five epoxy resin stumps simulating upper molars were fabricated to carry complete coverage crowns. Forty-five full coverage ceramic-fused-to-metal crowns with a non-precious metal alloy core (Co-Cr, Wirobond C®, Bego, Bremen, Germany) and ceramic covering (IPS d.SIGN, Ivoclar Vivadent®, Schaan, Liechtenstein) were fabricated, together with a further 30 crowns with an IPS e.maxZirCAD (Ivoclar Vivadent®) zirconia core and ceramic covering (IPS e.maxCeram, IvoclarVivadent®). All crowns were bonded with a self-curing luting composite (Multilink ® Automix, Ivoclar Vivadent®). Then, samples were subjected to static loading with a compression testing machine (Shimadzu model AG-X plus, Shimadzu Corporation, Kyoto, Ja-pan) until adhesive fracture of the ceramic covering occurred. The fractured crowns were then divided randomly into groups according to the core material and the surface preparation to be used prior to repair by composite resin.

• Ceramic-fused-to-metal crowns.

- Group I (n=15): Macro-retention was created on the exposed surface of the metal core with a circular diamond bur and the covering ceramic was treated with 9.5% hydrofluoric acid (Porcelain Etch, Ultradent® Products Inc., South Jordan, UT, U.S.A.) for two minutes.

- Group II (n=15): Air-particle abrasion of the crown core was carried out with 50µm aluminum oxide particles (Al2O3) using an intra-oral air abrasion device (Cojet Prep, 3M ESPE, CoJet System® 3M ESPE, Seefeld, Germany) from a distance of approxi-mately 20mm at a pressure of 2-3 bars for 10 seconds. Afterwards, the covering ceramic was etched with 9.5% hydrofluoric acid for 2 minutes (Porcelain Etch, Ultradent®).

- Group III (n=15): Samples were silica-coated with 30µm silica-coated (SiO2) aluminum oxide particles using an intraoral abrasion device (Cojet Prep, 3M ESPE) at a pressure of 2-3 bars from a distance of 20mm for 15 seconds.

Zirconia crowns.

- Group IV (n=15): air-particle abrasion of the crown core was carried out with aluminum oxide particles (Al2O3) as in Group II.

- Group V (n=15): Samples were silica-coated with 30 µm silica-coated (SiO2) aluminum oxide particles as in Group III.

After surface treatment, all the fractured crowns were repaired using a composite-based intra-oral repair system (Clearfil Repair, Kuraray Co. Ltd., Osaka, Japan) following the manufacturer’s instructions as follows: 1. 37% orthophosphoric acid (for 5 seconds); 2. Samples were washed in water and dried; 3. A metal primer was applied (Alloy Primer®. Kuraray); 4. A silane coupling agent (Clearfil Porcelain Bond Activator) and self-etching primer (Clearfil Se Bond Primer) were applied for 5 seconds; 5. The adhesive was applied (Clearfil Se Bond Bond); 6. Samples were air-dried and polymerized for 10 seconds; 7. Composite resin (Clearfil ST opaque) was placed on the exposed metal cores and polymerized for 40 seconds; 8. Composite resin (Clearfil AP-X C3) was placed and polymerized for 40 seconds building up the repair gradually until the restoration was complete.

When the samples had been repaired, they were subjected to dynamic loading in a chewing simulator (SD Mechatronik, Chewing Simulator CS-4. Mechatronik GmbH, Feldkirchen, Germany); they received 120,000 cycles with loads of 80N and 2Hz frequency with a vertical displacement of 2mm. They then underwent thermocycling (Thermocycler 2000, Heto-Holten A/S, Allerod, Denmark) for 6000 cycles with temperature changes between 5ºC and 55ºC every 30 seconds. When the samples had been fatigued, they were subjected to load testing (Shimadzu model AG-X plus) until the point of fracture, registering the final fracture values in Newtons (N), as well as the type of fracture produced.

-Statistical analysis: Differences between groups were analyzed by one-way Analysis of Variance (ANOVA) with repeated mea-sures and the Bonferroni test. The significance level was set at 5% (*p*=0.05%).

## Results

**Figure 1:**
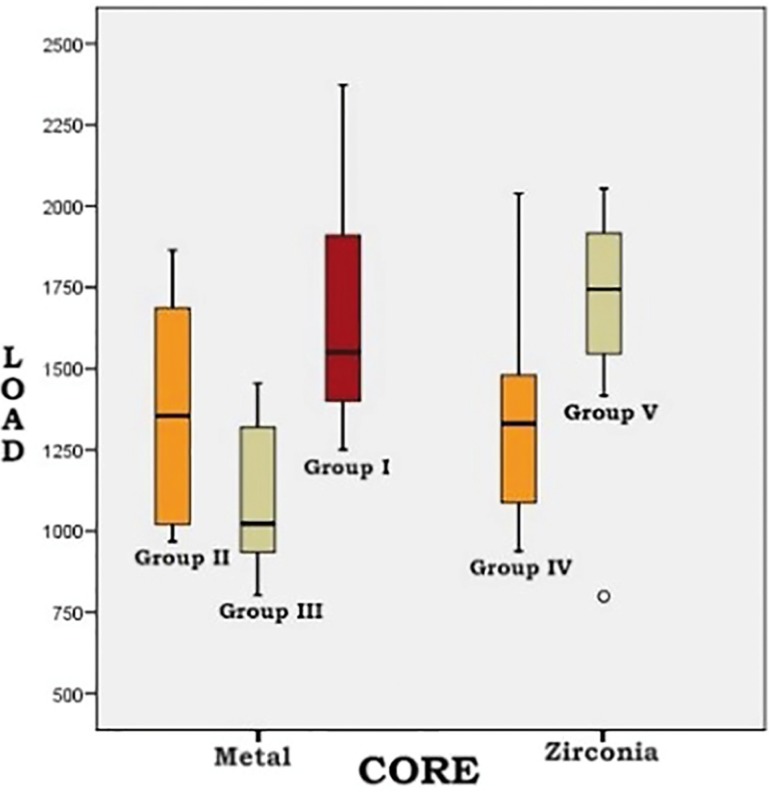
Box plot shows the distribution of fracture resistance values in relation to crown core material and surface preparation.

Repaired ceramic-fused-to-metal crowns obtained mean final fracture values of 1372.4N, Group I (diamond bur + HF) obtaining the highest value (1667.2±394.3N). Bonferroni bivariate contrast analysis revealed that mean fracture resistance was significantly higher for Group I than for Group III (silica-coated) (1094.3±212.6N) (*p*= 0.001). However, no evidence was found of higher fracture resistance in Group I than Group II (air-particle abrasion) (1355.6±333.8N)(*p*=0.137). No statistically significant differences were found between Group II (air-particle abrasion) and Group III (silica-coated) (*p*=0.275) (Fig. 1).

**Figure 2:**
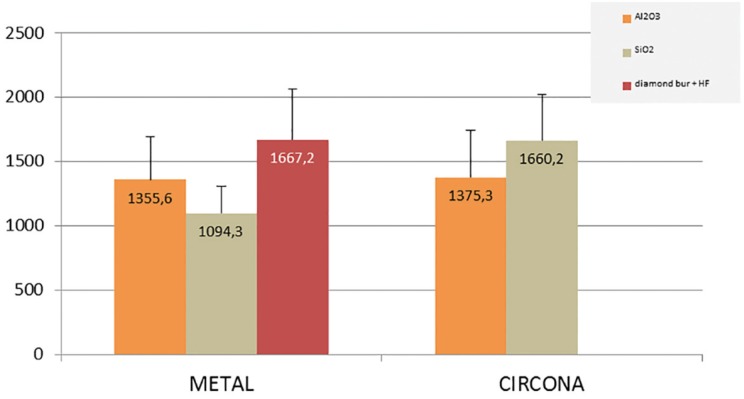
Mean fracture resistance (N) in relation to crown core material and surface preparation.

Although crowns with zirconia cores showed a tendency for samples repaired with silica-coating to obtain higher final fracture values (1660.2±361.6N, compared with an overall mean fracture resistance of 1517.8N), no significant differences were found between these samples (Group V) and samples treated with air-particle abrasion (Group IV: 1375±362.9N) (*p*=0.067) ( Table 1) (Fig. 2).

**Table 1:**
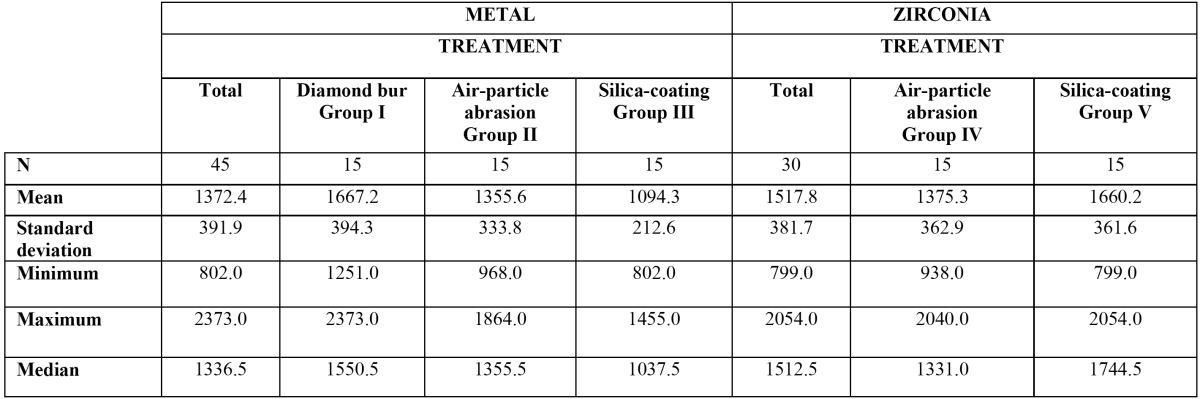
Resistance to fracture (N) of repaired specimens, analyzing the Crown core material variable and surface preparation method prior to repair.

**Figure 3:**
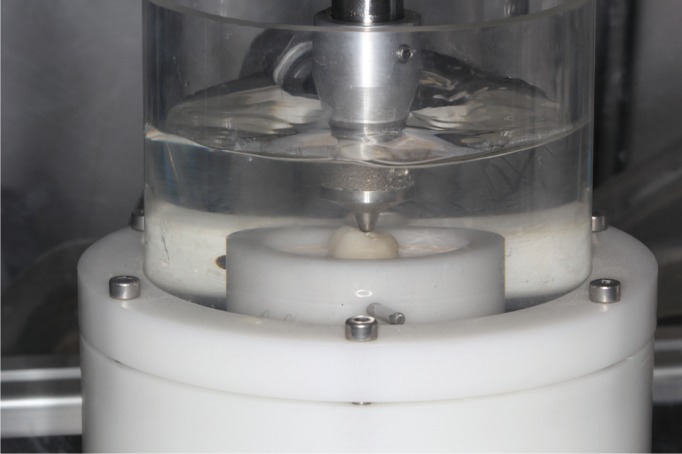
Sample undergoing dynamic loading and thermocycling.

Observing the type of fracture produced, all in vitro mechanical failures were found to be adhesive fractures (Fig. 3).

## Discussion

In vitro thermocycling and dynamic loading is a common method for testing dental materials to assess their suitability for in vivo use (5); the technique reproduces real conditions in the oral environment as faithfully as possible (6,7).

In the present study, ceramic-fused-to-metal crowns were seen to show higher final fracture values when the surfaces were prepared by creating macro-retentions with a diamond bur and by treating the ceramic covering with hydrofluoric acid (Group I: 1667.2±394.3N), but showed no significant difference in comparison with air abrasion with aluminum oxide particles (1355.6±333.8N). These data differ from other published findings, in which lower fracture values were obtained when surface preparation with a diamond bur was compared with air-particle abrasion and silica-coating (7,8). This is probably due to the fact that most of these studies only investigated the surface conditioning of a single material (metal or ceramic) (5-10).

Ceramic-fused-to-metal crowns prepared by silica-coating achieved the lowest final fracture values, a finding that coincides with studies such as that of Fonseca *et al.* (11). However, another study by Ozcan (7) found that silica-coating produced higher fracture values (582N) but without significant differences in comparison with surface preparation by air-particle abrasion (432N) or acid etching (376N) (11).

For zirconium oxide crowns, samples treated with silica-coating achieved higher final fracture values, although without significant differences in comparison with air-particle abrasion (50µm), a finding that coincides with other studies such as that of Attia *et al.* (5).

Statistical analysis identified a power of 0.68 for the effect contrast for surface treatment and 0.79 for the effect contrast of the core material and interaction (high-capacity of the statistical tests).

## Conclusions

1. For composite repairs to ceramic-fused-to-metal crowns, preparation of the metal core by diamond bur, conditioning the ceramic covering with hydrofluoric acid, produced significantly higher final fracture values than silica-coating.

2. For composite repairs to zirconia crowns, silica coating showed better performance than air-particle abrasion.

3. All fractures of the repaired specimens were adhesive fractures with complete detachment of the composite repair material.
